# Age-Related Variations in the Apparent Metabolizable Energy of Meat and Bone Meal for Broilers

**DOI:** 10.3390/ani14040530

**Published:** 2024-02-06

**Authors:** Mahmoud M. Khalil, M. Reza Abdollahi, Faegheh Zaefarian, Velmurugu Ravindran

**Affiliations:** 1 Monogastric Research Center, School of Agriculture and Environment, Massey University, Palmerston North 4442, New Zealand; 2 Animal Production Department, Faculty of Agriculture, Benha University, Benha 13736, Egypt; 3 Adisseo France S.A.S. European Laboratory of Innovation Science & Expertise (ELISE), Department of R&I in Monogastric Animal Nutrition, 20 Rue Prosper Monnet, 69190 Saint Fons, France

**Keywords:** age, apparent metabolizable energy, meat and bone meal, broilers

## Abstract

**Simple Summary:**

While regulations and guidelines vary globally and across nations, meat and bone meal (MBM) can be utilized for nutritional purposes for monogastric. Meat and bone meal is commonly included in poultry feed as a source of amino acids, calcium and phosphorus. The optimal inclusion of MBM in poultry diet formulation depends on the accurate estimation of the metabolizable energy content of MBM. The current available apparent metabolizable energy (AME) value of MBM is acquired from reference tables or equations based on assessments conducted with old birds (5 weeks old). This approach overlooks the potential influence of the age of broilers on the digestibility and utilization of energy-derived nutrients, hence AME or nitrogen-corrected AME (AMEn) values of MBM. Therefore, the present study aimed to examine the impact of the age of broilers on the AMEn of MBM from day 1 to 42 of age. The results revealed that the AMEn of MBM increased with the advancing age of broilers. Age-dependent AMEn values of MBM might be adopted in feed formulation for broilers.

**Abstract:**

The influence of broiler age on the apparent metabolizable energy (AME) and nitrogen-corrected AME (AMEn) of meat and bone meal (MBM) was investigated. A corn–soy basal diet and an experimental diet wherein 300 g/kg of the basal diet was replaced (*w*/*w*) with MBM were developed. The diets, in pellet form, were fed to six replicate cages across six age groups, namely d 1 to 7, 8 to 14, 15 to 21, 22 to 28, 29 to 35 or 36 to 42 d post-hatch. Birds were fed either a starter diet from d 1–21 or a finisher diet from d 22–35. Basal and experimental diets were introduced on d 1, 8, 15, 22, 29 and 36 with 10 (d 1–7), 8 (d 8–14) and 6 (d 15–42) birds per replicate. Total collection of excreta was carried out during the last 4 d of each age period. A linear decrease (*p* < 0.001) in the retention of dry matter and nitrogen was observed with advancing age. The AMEn of MBM showed a linear increase (*p* < 0.05), rising from 12.56 MJ/kg during d 1–7 to 13.90 MJ/kg during d 29–35, followed by a decline to 13.41 MJ/kg during d 36–42. The current findings showed that the energy utilization of MBM increased with the advancing age of broilers. Age-dependent AMEn values of MBM may need to be considered when MBM is included in feed formulations.

## 1. Introduction

Meat and bone meal (MBM) is a by-product of the rendering industry produced from mammalian tissue and bones and exclusive of any added hair, horn, hoof and blood [1]. It is widely used as a protein source in broiler diets due to its high protein content, amino acid profile and vital source of energy, calcium, phosphorus and other trace minerals [2,3]. However, the nutritional value of MBM may vary depending on several factors, such as the origin, differences in raw materials and the ratio between bone and soft tissues, processing conditions and the method of digestibility measurement [4,5,6].

The apparent metabolizable energy (AME) is a measure of the metabolizability of the energy in the feed and is calculated as the difference between the gross energy of ingested feed and the gross energy lost in the excreta [7]. The AME is a crucial parameter for formulating broiler diets, and it is the most common energy system used in diet formulation. Previous studies have reported variable AME values for MBM, ranging from 8.95 to 11.72 MJ/kg [3,8,9,10,11].

During the early stage of life, the digestion and utilization of nutrients are poor, especially during the first 10 days post-hatching. As broilers age, the digestive tract physiology goes through significant changes with rapid growth of the small intestine and an increase in the concentration of digestive enzymes [12,13]. Bird age has been reported to influence the digestion and absorption of energy-yielding nutrients in feed ingredients [14,15,16]. Adeola et al. [14] reported an increase in AMEn of MBM by 0.66 MJ/kg from 11.24 MJ/kg in week 1 to 11.90 MJ/kg in week 3.

As MBM is accepted by many countries as a feed ingredient for poultry, the accurate estimation of the AMEn of MBM and understanding the influence of broiler age on the AMEn of MBM is crucial for optimizing the use of MBM in broiler diets and reducing production costs. However, the influence of broiler age on the AMEn of MBM has not been extensively studied. Therefore, the aim of this study was to determine the AMEn content of MBM from 7–42 days of age for broiler chickens.

## 2. Materials and Methods

The experimental procedures were approved by the Massey University Animal Ethics Committee.

### 2.1. Ingredient

The meat and bone meal, of New Zealand origin, was procured from a local supplier. Representative samples of MBM were analyzed, in duplicate, for dry matter (DM), nitrogen (N), crude fat, neutral detergent fiber (NDF), ash, calcium (Ca), phosphorus (P) and gross energy (GE).

### 2.2. Birds, Diets and Housing

A total of 252, day-old male broilers (Ross 308) at 1 d of age were housed on floor pens prior to the weekly allocation to experimental diets. Birds were fed either a starter diet (230 g/kg crude protein and 12.56 MJ/kg AME) until d 21 or a finisher diet (207 g/kg crude protein and 13.0 MJ/kg AME) from d 22 to 35. A corn–soy basal diet (210 g/kg crude protein and 12.34 MJ/kg AME) was formulated as previously described in Khalil et al. [16], and the test diet was formulated by replacing (*w*/*w*) 300 g/kg of the basal diet with MBM. Diets were blended through a single-screw paddle mixer, and the mixed diet was pelleted using a pellet mill [16].

At the start of each week, birds were weighed individually and distributed to six replicate cages to ensure a consistent average body weight per cage. The basal and test diets were offered during the six periods, namely week 1, 2, 3, 4, 5 and 6, representing the growing periods d 1–7, d 8–14, d 15–21, d 22–28, d 29–35 and d 36–42, respectively. The number of birds per cage was 10, 8 and 6 for week 1, week 2 and weeks 3–6, respectively.

Birds were raised in an environmentally controlled house at 31 °C on d 1, and the temperature was gradually dropped to reach 22 °C by the end of week 3. The ventilation was controlled via extraction fans and wall inlet ducts. During the whole experimental period, feed and water were provided ad libitum.

### 2.3. Determination of Metabolizable Energy

The AME of MBM was determined following the substitution method and the total excreta collection [17]. During each week, fresh excreta were collected daily during the last 4 d, subsequently pooled by the cage and stored at −20 °C until freeze-dried. Dried samples were ground into a homogenous mixture through a 0.5 mm sieve and stored at 4 °C until laboratory analysis.

### 2.4. Chemical Analysis

The diet and excreta samples were analyzed for DM, GE and N using standard procedures. The MBM samples were analyzed for DM, GE, N, ash, crude fat, NDF and minerals. For DM determination, samples were analyzed according to the procedures of AOAC (Method 930.15) [18]. Gross energy was determined in an adiabatic bomb calorimeter (Gallenkamp Autobomb, Weiss Gallenkamp Ltd., Loughborough, UK) using benzoic acid as a calibration standard. Total N was assayed by combustion (Method 968.06) [18] using a carbon nanosphere-200 carbon, N and sulfur auto-analyzer (rapid MAX N exceed, Elementar, Donaustraze, Hanau, Germany). Crude protein was calculated by multiplying percentage N by a correction factor (6.25). Ash content was determined by Method 942.05 of AOAC [18] using a muffle furnace at 550 °C for 16 h. Fat content was determined by extracting fat in a Soxhlet extractor (Soxtec System HT 1043 Extraction Unit, Höganäs, Sweden) according to the AOAC procedure (Method 2003.06) [18]. The neutral detergent fiber was determined using Tecator Fibertec™ (FOSS Analytical AB, Höganäs, Sweden) following the standard procedure (Method 2002.04; AOAC, 2016) [18]. For mineral analysis, samples were first ashed and digested with HCl, and calcium and phosphorus contents were determined by Inductively Coupled Plasma–Optical Emission Spectroscopy (ICP-OES) using a Thermo Jarrell Ash IRIS instrument (Thermo Jarrell Ash Corporation, Franklin, MA, USA; Method 968.08D) [19].

### 2.5. Calculations

Dietary AME (MJ/kg DM) values were calculated by the following formulas described by Khalil et al. [16].

AME_Diet_ (MJ/kg) = [(Feed intake × GE_Diet_) − (Excreta output × GE_Excreta_)]/Feed intake (1)

The AME calculation of MBM (MJ/kg DM) according to the substitution method was determined as follows:

AME_MBM_ (MJ/kg) = [AME of MBM test diet − (AME of basal diet × 0.70)]/0.30 (2)

Nitrogen retention, as a percentage of intake, was determined as follows:

N retention (%) = 100 × [((Feed intake × N_Diet_) − (Excreta output × N_Excreta_))/(Feed intake × N_Diet_)] (3)

The AMEn (MJ/kg DM) was calculated by using the correction factor of 36.54 KJ per g N retained in the body, as suggested by Titus et al. [20].

### 2.6. Statistical Analysis

The data were subjected to one-way ANOVA analysis using the PROC GLM of SAS 9.4 (SAS Institute Inc., Cary, NC, USA) with the cage serving as the experimental unit. Significant differences between treatment means were determined by using the least significant difference test. Differences were considered significant at *p* < 0.05. All data were subjected to orthogonal polynomial contrasts using the GLM procedure of SAS version 9.4 [21] to examine whether responses to increasing bird age were of a linear or quadratic nature.

## 3. Results

The analyzed proximate and mineral composition of the test MBM is summarized in Table 1.

**Figure 1:**
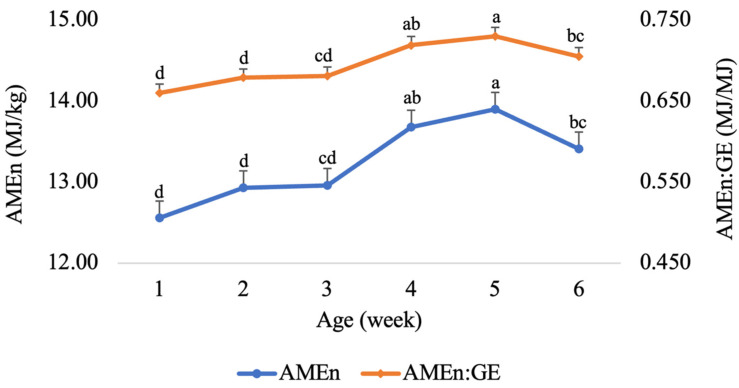
Effect of broiler age on the nitrogen-corrected apparent metabolizable energy (AMEn) and the ratio between AMEn and gross energy (GE) of meat and bone meal; mean ± standard error. a–d; values with different superscripts differ significantly (*p* < 0.05).

The influence of broiler age, from week 1 to 6, on the retention of DM and N, AME, AMEn and the ratio between AMEn and GE of MBM are summarized in Table 2. The retention of both DM and N decreased linearly (*p* < 0.001) as the broilers grew older. The highest DM and N retentions were recorded in week 1 and the lowest in week 6. The AMEn of MBM exhibited a linear response (*p* < 0.05) with the advancing age (Figure 1), with the AMEn increasing from 12.56 MJ/kg in week 1 to 13.90 MJ/kg in week 5. The AMEn:GE ratio numerically increased with advancing age of broilers, but the differences were not significant (*p* > 0.05).

The influence of broiler age on the GE of excreta and the ratio between excreta output to feed intake (FI) in birds fed MBM diet are summarized in Table 3. A quadratic response (*p* < 0.001) was observed for the effect of broiler age on the excreta GE. The excreta GE increased from 15.49 MJ/kg DM in week 1 to 15.89 MJ/kg DM in week 2 and then decreased in the following weeks. The ratio between excreta output and FI increased linearly (*p* < 0.001) as birds grew. Week 1 showed the lowest excreta output to FI ratio of 0.26 kg:kg, and the highest ratio of 0.35 kg:kg was recorded in week 6.

## 4. Discussion

The supply of optimum energy is the first factor considered in feed formulations, as changes in dietary energy content play a pivotal role in determining both diet cost and feed intake. Meat and bone meal is widely used as a low-cost protein source in poultry diets [3]. In addition to supplying amino acids, MBM is a good source of energy in broiler diets. Therefore, precise information on the available energy content of MBM is critical for optimizing the inclusion level of this ingredient in broiler diets.

The composition of the MBM sample evaluated in the current study is comparable to those reported by Adedokun and Adeola [3], Shi and Noblet [22], Wang and Parsons [23] and Ravindran et al. [24]. However, Adeola et al. [14] reported lower values of DM, CP and crude fat and higher values of GE, Ca and P. Sartorelli et al. [25] presented relatively lower values for CP and GE and higher values for ash, Ca and P. The variation in the composition of MBM samples could be explained by differences in the processing methods and the origin and source of the MBM [23,26,27].

The current practice in the formulation of diets is to apply a single AMEn value for the entire bird growth period regardless of broiler age. Therefore, the objective of the current study was to estimate the AMEn of MBM for broiler chickens at different ages, from weeks 1 to 6 of age. In an investigation by Adeola et al. [14], the AMEn of MBM was measured from weeks 1 to 3. To the authors’ knowledge, no published data are available on age-related AME values of MBM from week 1 to 6 post-hatch for broilers.

The current results showed that the AMEn of MBM increased with the advancing age of the broiler, where the lowest AMEn value was observed at the first week of age, and the highest AMEn content of MBM was at week 5, followed by a decrease in week 6. Similarly, the AMEn to GE ratio increased from week 1 to week 5 of age, followed by a decrease between weeks 5 and 6. However, the retention of DM and N declined linearly as the birds grew older.

A previous study from our laboratory [16] showed that the AMEn of two major protein sources, namely soybean meal and canola meal, was age-dependent and decreased quadratically with the advancing age of broilers. These findings are in contrast with those of the current study, which could be due to the differing nutritional composition of the dietary ingredients and the utilization of nutrients in different protein sources. In agreement with the current findings, Adeola et al. [14] showed that the AMEn of MBM increased from 13.10 MJ/kg in week 1 to 13.66 MJ/kg in week 3 for broilers. Similarly, the present findings are consistent with previous research conducted with broilers fed complete diets, reporting that the utilization of energy-yielding nutrients in diets improved as the age increased [28,29,30]. Aderibigbe et al. [31] showed that the AMEn of a corn–soy diet increased by 0.20 MJ/kg between 11 and 21 d of age, with no further changes until 42 d of age. Thomas et al. [32] observed that the AMEn of wheat- and maize-based diets increased from 11.06 to 13.24 MJ/kg and from 12.28 to 13.01 MJ/kg between d 7 and 14 of age, respectively. The increases observed in the AMEn of MBM could be explained by the development of digestive and absorptive capacities of the digestive tract of broilers with advancing age. The production of digestive enzymes and nutrient absorption capacity tends to increase as birds grow older; most probably, the ability of birds to extract nutrients from feeds improves with age. The increase in AMEn trends is to be expected in diets containing adequate amounts of protein, as the AME is a function of the use of energy-yielding components (lipids and starch).

While the current study did not assess the fat digestibility in MBM, existing evidence on fat digestion dynamics supports the observed increase in AMEn of MBM with advancing broiler age. Tancharoenrat et al. [33] examined the influence of the age of broilers on various fat sources and found that total tract fat digestibility was initially low in week 1 but increased as birds aged. Similarly, Lessire et al. [34], in their examination of the age-related effects on fat digestibility and AME of beef tallow, revealed an increase of 8.5% in the apparent fat digestibility and a 4.3% increase in AME between weeks 2 and 6. Scheele et al. [35] demonstrated an increase in the apparent digestibility of animal fat after week 2, with a corresponding 1.0 MJ/kg increase in the AME from week 2 to week 4.

In the current study, the AMEn:GE ratio for MBM increased with the advancing age of the broilers. In contrast, Moss et al. [36] reported that the AME:GE of a wheat-based diet was higher at 7 days of age than at 34 days of age (0.799 vs. 0.761). It appears that the energy metabolizability of different ingredients might behave differently to the age effect.

In the present study, the highest retention values of DM and N were recorded in week 1 and declined thereafter as birds grew older. Similarly, Khalil et al. [16] observed that the DM and N retention of SBM and CM diets decreased with advancing age, with the highest DM retention being recorded for week 1. The N retention decreased linearly by 25.8% between weeks 1 and 6 for SBM and by 27.5% between weeks 1 and 6 for CM. Similarly, in the current study, the N retention in birds fed the MBM diet decreased by 25.8% with the advancing age of broilers. Aderibigbe et al. [31] also found that the retention of DM and N for corn–SBM diet decreased with advancing age from d 1 and 42 of age for broilers. Yang et al. [37] reported a significant decrease in the retention of DM in cereals-based diet by 2.2% between d 28 to 35 d of age.

The higher retention values of DM and N estimated at week 1 are counter-intuitive, as newly hatched chicks have immature digestive tracts with low secretion and activities of the endogenous digestive enzymes [38,39]. A study by Uni et al. [40] showed that the retention of N increased with advancing age from 70 to 90% between d 4 and 14 post-hatch. Olukosi et al. [41] revealed that the retention of both DM and N increased by 20% between weeks 1 and 2 post-hatch.

The possible explanation for the reduction of DM and N retention with age could be the low FI in the first days of age post-hatch. A low FI reduces the digesta passage rate in the digestive tract, which improves the digestibility of nutrients as it allows for prolonged interaction with the digestive enzymes and absorptive cells [42]. Vergara et al. [43] revealed that the increase in digesta passage rate is associated with the increase in FI with advancing age. Uni et al. [40] reported that the digesta passage time in the digestive tract increased from 74 min to 122 min at d 7 and d 22 of age, respectively.

Using a single AMEn estimate of MBM for all ages will lead to overestimation or underestimation of the available energy content of MBM and the energy requirement of broilers, thereby impacting the accuracy of feed formulations. Being the first study reporting the AMEn estimates of MBM over the whole broiler growth cycle, this is a timely addition to the currently existing AMEn estimates database. It presents an opportunity to employ age-specific AMEn values of MBM for broilers, eliminating the risk of inaccuracies in different growth stages and enhancing the precision of feed formulations and the sustainability of broiler production. Nonetheless, further research is necessary to compare the growth performance and economic returns of feeding broiler chickens with diets formulated based on the current AMEn values of MBM and those derived from the current study.

## 5. Conclusions

The current findings demonstrate that the age of broilers has a significant influence on the AME and AMEn of MBM. The AMEn of MBM increased with advancing age, with the lowest AMEn value at week 1 and the highest AMEn value at week 5. The present findings indicate that relying on a single AME or AMEn value for MBM in diet formulation for broilers is debatable. Age-dependent AME or AMEn values should be taken into account when formulating feed to optimize the utility of MBM and the economic returns.

## Figures and Tables

**Table 1:** Proximate and mineral composition of meat and bone meal (g/kg; as received basis).

Item	Meat and Bone Meal
Dry matter	968
Ash	149
Nitrogen (N)	103
Crude protein (N × 6.25)	644
Crude fat	142
Neutral detergent fiber	154
Calcium	41.3
Phosphorus	23.3
Gross energy (MJ/kg)	21.19

**Table 2:** Influence of broiler age on the retention (% of intake) of dry matter (DM) and nitrogen (N), apparent metabolizable energy (AME; MJ/kg DM), nitrogen-corrected AME (AMEn; MJ/kg DM) and the ratio between AMEn and gross energy (GE; MJ/MJ) of meat and bone meal ^1^.

Age (Week)	DM Retention	N Retention	AME	AMEn	AMEn:GE
1	73.7	63.5	14.62	12.56	0.660
2	72.2	61.6	14.94	12.93	0.679
3	68.5	54.9	14.60	12.96	0.681
4	69.6	56.6	15.51	13.68	0.719
5	69.2	53.5	15.58	13.90	0.730
6	65.6	47.2	15.22	13.41	0.705
SEM ^2^	0.58	1.02	0.265	0.164	0.009
Orthogonal polynomial contrast, *p*≤					
Linear	0.001	0.001	0.059	0.002	0.188
Quadratic	0.571	0.449	0.733	0.672	0.538

^1^ Each value represents the mean of six replicates. The number of birds per replicate cage was 10 (week 1), 8 (week 2) and 6 (weeks 3–6). ^2^ Pooled standard error of the mean.

**Table 3:** Influence of broiler age on excreta gross energy (GE; MJ/kg DM) and excreta output:feed intake (kg:kg) in birds fed the meat and bone meal diet ^1^.

Age (Week)	Excreta GE (MJ/kg DM)	Excreta Output:Feed Intake
1	15.49	0.26
2	15.89	0.28
3	15.55	0.32
4	15.13	0.31
5	14.93	0.31
6	14.47	0.35
SEM ^2^	0.079	0.006
Orthogonal polynomial contrast, *p*≤		
Linear	0.001	0.001
Quadratic	0.001	0.633

^1^ Each value represents the mean of six replicates. The number of birds per replicate cage was 10 (week 1), 8 (week 2) and 6 (weeks 3–6). ^2^ Pooled standard error of the mean.

## Data Availability

All available data are incorporated in the manuscript.
